# Antipsychotic-induced catatonia and neuroleptic malignant syndrome: the dark side of the moon

**DOI:** 10.1038/s41380-021-01158-2

**Published:** 2021-05-19

**Authors:** Dusan Hirjak, Alexander Sartorius, Katharina M. Kubera, Robert Christian Wolf

**Affiliations:** 1 Department of Psychiatry and Psychotherapy, Central Institute of Mental Health, Medical Faculty Mannheim, University of Heidelberg, Mannheim, Germany; 2 Center for Psychosocial Medicine, Department of General Psychiatry, University of Heidelberg, Heidelberg, Germany

**Keywords:** Neuroscience, Schizophrenia

## To the Editor:

We were very pleased to read the excellent umbrella review (UR) by van der Burg et al. [1] who highlighted genetic factors associated with drug-related movement disorders (DRMD) such as tardive dyskinesia (TD), parkinsonism, akathisia, and acute or tardive dystonia. The authors reviewed and discussed 15 meta-analyses reporting on genetic variations in ten genes, including DRD3, DRD2, CYP2D6, HTR2A, COMT, HSPG2, and SOD2. The authors concluded that these gene variants may be particularly relevant for DRMD. The authors have to be commended for their timely and superb work. Nevertheless, we do believe their UR is incomplete in that it did not consider the antipsychotic-induced catatonic symptoms (ACS) and neuroleptic malignant syndrome (NMS) [2]. In daily clinical routine, any sensorimotor deterioration in psychiatric patients on antipsychotics is readily attributed to NMS or TD, neglecting the patients’ history. Also, many clinicians may not be fully aware of the risk of developing ACS or NMS. Although ACS and NMS are epidemiologically rare events, they are clinically highly relevant DRMD since they are frequently associated with potentially life-threatening complications. Therefore, clinicians should consider genuine catatonia or DRMD (e.g., ASC, NMS, and TD) whenever novel sensorimotor signs occur or present sensorimotor symptoms are deteriorated. Identifying susceptibility genes for ACS and NMS could foster prevention of these conditions as much as it could contribute to personalized treatment decisions, particularly with respect to the choice of a specific antipsychotic drug.

It is essential to be aware that the vast majority of studies have focused on genuine catatonic symptoms [3] and ACS are often neglected or unknown in daily clinical practice. Stubner et al. [4] reported five cases of ACS in a group of 86,439 patients with antipsychotic treatment, but the exact incidence of ACS is still unknown. Previous case reports have described the occurrence of ACS within hours after the first administration of both first-generation antipsychotics (FGA) and second-generation antipsychotics (SGA) [2]. Patients with affective (e.g., extreme anxiety and affect incontinence) and behavioral catatonic symptoms (e.g., impulsiveness and aggressiveness) may develop ACS if incorrectly diagnosed with mania and treated with intravenous or intramuscular FGA. Furthermore, treatment with FGA can lead to further deterioration of akinesia, rigor, mutism, and rigid affect [5]. Given some overlapping signs and symptoms, it is often difficult to reliably disentangle genuine catatonia from ACS in clinical practice. While akinesia, stupor, and mutism (akinetic mutism) are common in ACS [6, 7], more complex motor or behavioral catatonic symptoms such as catalepsy, waxy flexibility, echolalia, echopraxia, verbigeration, or “mitgehen” have not been reported in the context of antipsychotic use (perhaps because such phenomena may have been overlooked) and might be rather seen in genuine catatonia [5].

Treatment of ACS (stopping or switching antipsychotic treatment) must be prompt to avoid further deterioration in the sense of malignant catatonia or NMS [8]. With regard to the arguments above, it is conceivable that the occurrence of undesirable DRMD may lead to a worsening of the primary disease and thus to an increase in ACS.

Despite the outstanding clinical relevance, evidence regarding susceptibility genes for ACS is very scarce. A simple search in PubMed on March 11th, 2021 using the terms “*catatonia*” and “*genes*” OR “*genetic*” identified a total of 211 results. For instance, two earlier studies by Stöber et al. [9, 10] reported susceptibility loci for “periodic catatonia” on chromosome 15q15 and 22q. Later on, Hagemeyer et al. [11] found that a myelin-associated gene CNP (2′,3′-cyclic nucleotide 3′-phosphodiesterase) co-determines the occurrence of a catatonia-depression syndrome upon aging. Interestingly, susceptibility regions on chromosome 15q15–q21 are shared between autism and catatonia [12]. In line with this, Peter-Ross [13] postulated aberrant SNORD115-1 (also called HBII-52) genes (either duplications or deletions) located on chromosome 15q11–13 (AUTS4) in the pathophysiology of catatonic symptoms in various neuropsychiatric disorders (e.g., Prader–Willy syndrome, Angelman syndrome, autism, NMDAR antibody encephalitis, or NMS).

NMS (e.g., hyperthermia, extrapyramidal signs, rhabdomyolysis, altered consciousness, and autonomic dysfunction) is a rare and life-threatening manifestation of DRMD that requires an efficient and timely therapy [14]. The scientific evidence is controversial, as information on symptoms, prevalence, course of disease and prognosis of NMS is mainly based on individual case reports and only a few reviews. In antipsychotic users, the incidence of NMS is ~0.06–1.4% [15]. Recent studies have shown that NMS is less pronounced among SGA compared to FGA (lethality is higher: 3% for FGA vs. 16.3% for SGA) [16, 17]. A simple search in PubMed on March 11th, 2021 using the terms “*neuroleptic malignant syndrome*” and “*genes*” OR “*genetic*” identified a total of 100 results. An earlier study by Suzuki et al. [18] postulated an association between NMS and *Taq*I A polymorphism of the dopamine D_2_ receptor (DRD2). Later on, Kishida et al. [19] found that the −141C Ins/Del (but not *Taq*I A [20] or Ser311Cys) polymorphism of the DRD2 is associated with NMS. Finally, there is preliminary evidence based on case studies for an association between CYP2D6 DRD2 polymorphism (with reduced function) and NMS.

In daily clinical practice, catatonic syndromes and ACS are primarily treated with benzodiazepines and electroconvulsive therapy (ECT) [5]. According to a recent systematic review [14], NMS should also be treated first line with dantrolene/bromocriptine or ECT. Since ACS and NMS respond excellently to ECT, a common genetic component is suggestive. In summary, both ACS and NMS belong to the group of DRMD because they are not only clinically related, but also show some overlapping gene polymorphisms (e.g., DRD2 and CYP2D6; similar to van der Burg et al. [1]). From a pathophysiological point of view, there are three potential pathomechanisms that may lead to rigor, stupor, and related sensorimotor/autonomic signs, which are characteristic symptoms of both ACS and NMS: (1) genuine catatonia can change into potentially life-threatening pernicious or malignant catatonia, (2) antipsychotic induced sensorimotor abnormalities such as parkinsonism may develop within days and weeks after titrating antipsychotic medication. These patients may also exhibit characteristic sensorimotor abnormalities such as akinesia, rigor, tremor, and related sensorimotor/autonomic signs [21], and (3) genuine catatonia may also progress to potentially life-threatening NMS when novel antipsychotic is commenced. From a clinical point of view the differential diagnosis of NMS vs. pernicious or malignant catatonia is often a challenge due to similar symptoms. For these particular reasons, psychiatric history taking, clinical blood tests, and gathering of information on the response to antipsychotics are crucial. Still, the distinction between these three phenomenologically very similar syndromes may be very complex. Therefore, clinical rating scales and standardized instrumental assessments should be used to measure sensorimotor dysfunction in psychiatric patients [22, 23]. When possible, as many different constructs (dyskinesia, parkinsonism, catatonia, akathisia, and NMS, etc.) as possible should be studied using different units of analysis within transdiagnostic study samples [22, 23].

The conclusion that can be drawn from the data is that both ACS and NMS have been clearly underappreciated by genetic research. Both ACS and NMS are clinically rather rare conditions, so that finding candidate genes that are associated with these disorders will require large groups of patients. Given the clinical relevance of these syndromes, we strongly endorse population-based cohort studies that could decisively contribute to the specification of risk (or protective) factors (including relevant gene variants) and predictors for the occurrence of ACS and NMS [22]. In line with this, we would appreciate hearing the authors’ opinion on the relationship between candidate genes and ACS and NMS.
